# Attitudes and stressors related to the SARS-CoV-2 pandemic among emergency medical services workers in Germany: a cross-sectional study

**DOI:** 10.1186/s12913-021-06779-5

**Published:** 2021-08-21

**Authors:** Annegret Dreher, Frank Flake, Reinhard Pietrowsky, Adrian Loerbroks

**Affiliations:** 1 Institute of Occupational, Social and Environmental Medicine, Centre for Health and Society, Faculty of Medicine, University of Duesseldorf, Duesseldorf, Germany; 2 German Association of Emergency Medical Service, Luebeck, Germany; 3 Institute of Experimental Psychology, Department of Clinical Psychology, University of Duesseldorf, Duesseldorf, Germany

**Keywords:** Emergency medical services workers, COVID-19, Cross-sectional study, Epidemiology, Occupational health, Psychological stress

## Abstract

**Background:**

The aim was to investigate attitudes and stressors related to the SARS-CoV-2 outbreak among emergency medical services (EMS) workers in Germany. We further aimed to detect possible changes within a 5-week period and potential determinants of attitudes and stressors.

**Methods:**

We conducted two cross-sectional studies using an online questionnaire in early April 2020 (i.e., the first peak of the SARS-CoV-2 outbreak in Germany) and five weeks later. The study instrument comprised sociodemographic items, self-devised items on pandemic-related attitudes, stressors and work outcomes, and established instruments assessing depressive symptoms and symptoms of anxiety. Logistic regression was performed to identify possible determinants.

**Results:**

Data of 1537 participants was included in the analysis (April: *n* = 1124, May: *n* = 413, 83.1% male, median age 32). Most participants agreed that their personal risk of infection was higher compared to the general population (April: 87.0% agreement, May: 78.9%). The greatest stressor was uncertainty about the pandemic’s temporal scope (82.0 and 80.9%, respectively). Most participants (69.9, 79.7%) felt sufficiently prepared for the pandemic and only few felt burdened by their financial situation (18.8, 13.3%). Agreement to all stressors decreased from April to May except related to the childcare situation. Regression analysis identified subgroups to be burdened more frequently such as older employees, those with SARS-CoV-2 cases among their colleagues, and those with lower paramedic training levels.

**Conclusions:**

We identified key SARS-CoV-2-related stressors whose levels generally decreased within a 5-week period. Our results indicate that EMS workers are less affected by existential fears and rather worry about their personal infection risk.

**Supplementary Information:**

The online version contains supplementary material available at 10.1186/s12913-021-06779-5.

## Background

The 2020 global SARS-CoV-2 pandemic posed great challenges to healthcare systems worldwide resulting in over 80,300,000 infections and claiming 1,770,000 deaths by the end of the year [1]. In Germany, as many as 1,650,000 cases have been confirmed with over 44,000 cases in need for intensive medical treatment [1, 2]. Emergency medical services (EMS) workers are in charge of pre-hospital emergency medical care. Due to close patient contact they are at high risk of contracting infectious diseases [3]. EMS workers may also be the first contact person for patients who suffer severely from COVID-19. These patients may have not yet been diagnosed with SARS-CoV-2 and therefore contact with those patients represents a high risk of transmission. Lindsley et al. (2019) found that aerosols resulting from patient coughs spread homogenously across the entire ambulance due to ventilation systems and that there are no spots with lower risk of infection within a vehicle [4].

Since the rescue service is an essential pillar of the health care system, several studies have already dealt with the question of how EMS workers are able to work in the best possible way when facing a pandemic situation. A recent study among EMS workers in Jordan found slightly more than two thirds of workers to feel adequately trained and knowledgeable about a possible infectious disease outbreak [5]. Yet, two thirds of participants were concerned about becoming infected and over 70% were concerned about a lack of personal protective equipment (PPE). A US study by Ventura et al. (2020) addressing the current SARS-CoV-2 outbreak found that 36% of EMS personnel had not received pandemic-specific training and half of the personnel was dissatisfied with the training they had received. Less than half of the surveyed personnel had access to N95 masks and in over 30% of cases masks were only exchanged once a week [6]. EMS workers’ presumed willingness to work during a pandemic has also been investigated in different studies and found to strongly depend on the availability of PPE, the opportunity of vaccination, the provision of pharmaceuticals such as pre exposure prophylaxis, and the probability of infecting own family members [7–9]. Much also depends on the employer: Rebmann et al. (2020) found that over 60% of EMS personnel in the US believed in their employer to take precautions and supply them with PPE in case of a pandemic [8]. Another study found more than half of EMS personnel to believe their employer had efficient systems to manage a possible outbreak and would update them with information [5]. In summary, the published literature suggests a moderate degree of perceived preparedness among EMS workers for infectious disease outbreaks with major concerns being the risk of infection and the lack of PPE.

Initial studies among EMS workers during the SARS-CoV-2 pandemic have investigated personnel’s knowledge about transmission routes and hygiene measures [10], possible correlates of stress [11], and the level of anxiety among EMS staff [12]. To our knowledge, only one study has so far shed light on EMS workers during the current SARS-CoV-2 pandemic in Germany. In their sample of over 2000 healthcare professionals, including 221 paramedics, Skoda et al. (2020) stated that these suffered from generalized anxiety disorder less often than physicians and nurses and had the best health status among these three professional groups [13]. Yet, neither this study nor others have yet investigated a broad range attitudes and stressors related to the current SARS-CoV-2 pandemic among EMS workers. The investigation of stressors is crucial as stress may lead to poor mental health resulting in a decreased quality of patient care [14, 15].

Our study consequently firstly aimed to address a broad scope of attitudes, stressors, and work outcomes for EMS workers during the SARS-CoV-2 pandemic. We aimed to characterize outcomes addressed in previous studies (e.g., feeling (in-)sufficiently prepared [5, 6], being concerned about an own infection [5], or concerned about a lack of PPE [6]), but also to characterize novel stressors that have not been investigated so far, such as one’s childcare situation, uncertainty about one’s financial situation or uncertainty about contact persons to get further information from. To the best of our knowledge, no studies have yet longitudinally investigated the development of stressors during a pandemic either. The second aim of this study was therefore to detect possible changes in attitudes and stressors during the SARS-CoV-2 pandemic within a 5-week period. The final aim of the study was to detect possible determinants in order to identify subgroups at increased risk of feeling burdened. Again, we aimed to include determinants that have not been investigated in this context before but can nevertheless be assumed to be relevant such as the need to care for underage children and the exact level of prior paramedic training.

## Methods

### Study setting and population

In 2019, there were over 71,000 EMS workers in Germany who mostly completed one of four training programs: Simple tasks such as driving the ambulance and assistance activities in patient care may be performed after 320 h of training (German profession: “Rettungshelfer”). A more extended training of 520 h (Emergency Medical Technician, German profession “Rettungssanitäter”) allows performing simple assistance activities and providing sole care of patients who are not vitally endangered. The highest possible non-medical training is completed after 3 years (Paramedic, German profession “Notfallsanitäter”) and allows for acting on sole responsibility. Formerly (up to 2014), only 2 years of training were required to perform on sole responsibility (profession “Rettungsassistent”) [16, 17]. All EMS workers of legal age (18 and older) and from all over Germany were eligible for inclusion to participate in our study.

### Study design

The German Association of Emergency Medical Service (Deutscher Berufsverband Rettungsdienst e.V.) published a 29-item online questionnaire on their social media channels between April 9th and April 16th, 2020 (first wave) and again between May 14th and May 21st (second wave). Over 9500 members are affiliated with the association across entire Germany. The questionnaire nevertheless had the potential to reach all EMS workers employed in Germany.

### Key outcome measures

**Fig. 1:**
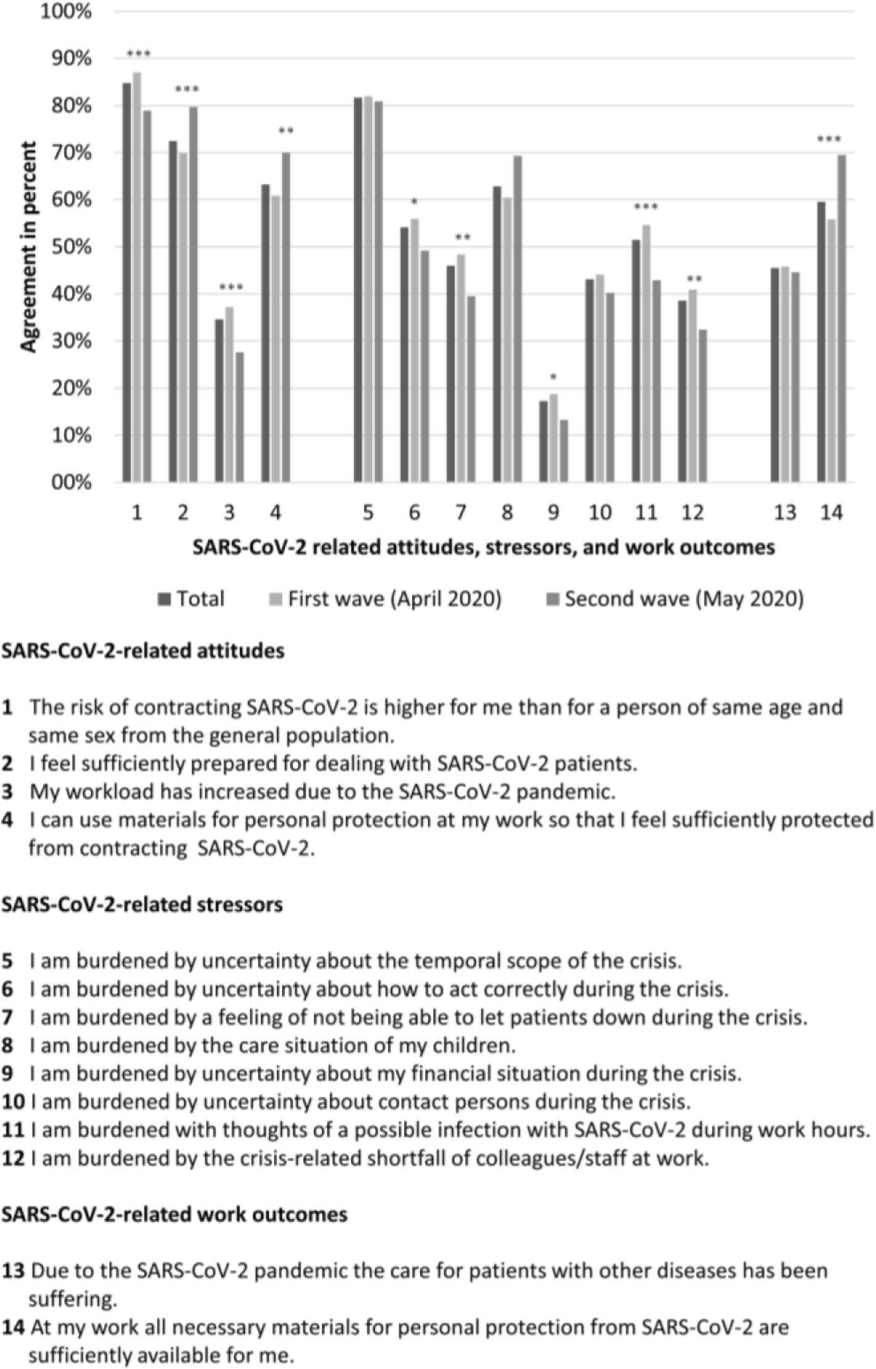
Items measuring SARS-CoV-2-related attitudes, stressors, and work outcomes

The study questionnaire consisted of three sections (see Additional file 1 for the scope and wording of items):

1. The first section covered socio-demographic characteristics, work-related characteristics, and questions on suspected or confirmed SARS-CoV-2 cases among either family and friends, colleagues, or oneself.
2. The second section included self-devised questions on SARS-CoV-2-related attitudes, stressors, and work outcomes (see Fig. 1 for scope and wording of items).
3. The third section contained questions on symptoms of depression and anxiety as measured by the validated 2-item measures of the Patient Health Questionnaire (PHQ-2) and the Generalized Anxiety Disorder questionnaire (GAD-2), respectively.

Wording of items measuring SARS-CoV-2-related attitudes, stressors, and work outcomes and percentage of agreement among 1537 EMS workers in Germany at two timepoints and in total. Fisher’s exact test *p < 0.05, **p < 0.01, ***p < 0.001.

The questionnaire published in May (second wave) furthermore included an item asking for whether participants had already participated in April. The development of items for the second section was based on published items measuring attitudes of medical staff during past infectious disease outbreaks [18–20] and on repeated discussion with experts of the German Association of EMS. These discussions covered the comprehensibility of questionnaire items for the target group, the appropriate questionnaire length, and the completeness of content. Experts of the association have not only worked as EMS workers themselves for many years but are in regular contact and exchange with EMS workers all over Germany through educational events, phone calls and own previous surveys among members. The answer options were provided using a four-point Likert Scale ranging from “Do not agree at all” to “Strongly agree”. The questionnaire was delivered using UNIPARK software.

### Data analysis

We ran descriptive analysis for all variables displaying absolute numbers and percentages and Fisher’s exact test to compare participants’ characteristics between the first and second wave. Fisher’s exact test was also run to determine any significant differences between prevalences of attitudes, stressors, and work outcomes of both study waves (see Fig. 1, *p < 0.05, **p < 0.01, ***p < 0.001). Including only data from the first study wave, we used a priori designed logistic regression models to identify possible associations between the items of questionnaire section two with those of section one and three. Due to the solely exploratory nature of this study no correction for multiple testing was done.

The original 4-point answer scales of SARS-CoV-2 related attitudes, stressors and work outcomes were dichotomized into answer options 1 “agree” and 0 “disagree”. Alternatively, ordinal regression analysis could have been performed which does not require dichotomization of scales and therefore prevents possible loss of information. However, for reasons of comprehensibility and due to the exploratory nature of our study, it seemed sufficient and more relevant to know whether participants feel burdened rather than the exact degree of burden. For PHQ-2 and GAD-2, the established cut-off values of ≥3 were used to classify participants into having depressive symptoms or a symptoms of anxiety [21, 22]. We ran two models for SARS-CoV-2 related attitudes and stressors: A first model only included age and sex as independent variables, a second, multivariable model contained all items from questionnaire section one and three. For SARS-CoV-2 related work outcomes only age, sex and paramedic training were included in the multivariable model. In sensitivity analysis, we omitted depression and anxiety (questionnaire section 3) from the multivariable model to reduce the likelihood that associations are due to negative affect. We ran further sensitivity analysis by pooling data from both study waves for logistic regression. Associations were reported as odds ratios (ORs) with respective 95% confidence intervals (CI). We ran all analysis using IBM SPSS Statistics 25.

### Outcomes of investigation

Outcomes in terms of SARS-CoV-2 related attitudes were:

1. I feel sufficiently prepared for dealing with SARS-CoV-2 patients (agree/disagree).
2. The risk of contracting SARS-CoV-2 is higher for me than for a person of same age and sex from the general population (agree/disagree).
3. At my work all necessary materials for personal protection from SARS-CoV-2 are sufficiently available for me (agree/disagree).
4. I can use materials for personal protection at my work so that I feel sufficiently protected from contracting SARS-CoV-2 (agree/disagree).

The following SARS-CoV-2 related stressors were investigated:

5. I am burdened with thoughts of a possible infection with SARS-CoV-2 during work hours (agree/disagree).
6. I am burdened by the crisis-related shortfall of colleagues/staff at work (agree/disagree).
7. I am burdened by the care situation of my children (agree/disagree).
8. I am burdened by uncertainty about how to act correctly during the crisis (agree/disagree).
9. I am burdened by uncertainty about contact persons during the crisis (agree/disagree).
10. I am burdened by uncertainty about my financial situation during the crisis (agree/disagree).
11. I am burdened by uncertainty about the temporal scope of the crisis (agree/disagree).
12. I am burdened by a feeling of not being able to let patients down during the crisis (agree/disagree).

SARS-CoV-2 related work outcomes included:

13. My workload has increased due to the SARS-CoV-2 pandemic (agree/disagree).
14. Due to the SARS-CoV-2 pandemic the care for patients with other diseases has been suffering (agree/disagree).

## Results

### Descriptive analysis

A total of 1675 EMS workers participated in the study. After removal of persons with missing data (*n* = 8) and those who reported participation in both waves (*n* = 130, removed due to a too small sample size for separate analysis), the final study sample comprised 1537 participants who had either participated in the first (*n* = 1124) or the second study wave (*n* = 413) (two independent samples). The characteristics of the study sample are displayed in Table 1. Median participant age was 32 years (interquartile range 28-37) and 83.1% of participants were male. As much as 15.3% of workers screened positive for depressive symptoms and 16.1% for symptoms of anxiety. Significant differences of participant characteristics between the first and second wave were found only for suspected or confirmed SARS-CoV-2 cases among colleagues with slightly less cases in the second wave (*p* = 0.01).

**Table 1:** Socio-demographic characteristics of *n* = 1537 study participants

Characteristics	Total (***n*** = 1537)	First wave (***n*** = 1124)	Second wave (***n*** = 413)	***p***-value
	***n*** (%)	***n*** (%)	***n*** (%)	
Sex				0.15
Male	1278 (83.1)	924 (82.2)	354 (85.7)	
Female	257 (16.7)	199 (17.7)	58 (14.0)	
Non-binary*	2 (0.1)	1 (0.1)	1 (0.2)	
Age, median (interquartile range)	32 (28-37)	32 (28-37)	32 (27-37)	0.77
18-28	546 (35.5)	394 (35.1)	152 (36.8)	
29-37	503 (32.7)	373 (33.2)	130 (31.5)	
38 and older	488 (31.8)	357 (31.8)	131 (31.7)	
Permanent Partner				0.64
Yes	1158 (75.3)	843 (75.0)	315 (76.3)	
No	379 (24.7)	281 (25.0)	98 (23.7)	
Children under care in same household				0.95
Yes	427 (27.8)	313 (27.8)	114 (27.6)	
No	1110 (72.2)	811 (72.2)	299 (72.4)	
Highest level of education				0.82
Low^1^	81 (5.3)	56 (5.0)	25 (6.1)	
Intermediate^2^	618 (40.2)	455 (40.5)	163 (39.5)	
High^3^	831 (54.1)	608 (54.1)	223 (54.0)	
Other	7 (0.5)	5 (0.4)	2 (0.5)	
Highest level of paramedic training				0.83
520 h training^a^	311 (20.2)	226 (20.1)	85 (20.6)	
2 years training^b^	143 (9.3)	109 (9.7)	34 (8.2)	
3 years training^c^	1065 (69.3)	776 (69.0)	289 (70.0)	
Other	18 (1.2)	13 (1.2)	5 (1.2)	
Self-rated health				0.50
Very good	459 (29.9)	329 (29.3)	130 (31.5)	
Good	948 (61.7)	704 (62.6)	244 (59.1)	
Moderate	125 (8.1)	88 (7.8)	37 (9.0)	
Bad	5 (0.3)	3 (0.3)	2 (0.5)	
Very bad	0 (0.0)	0 (0.0)	0 (0.0)	
Suspected or confirmed SARS-CoV-2 cases among friends and family				0.37
Yes	284 (18.5)	214 (19.0)	70 (16.9)	
No	1253 (81.5)	910 (81.0)	343 (83.1)	
Suspected or confirmed SARS-CoV-2 cases among colleagues				0.01**
Yes	842 (54.8)	638 (56.8)	204 (49.4)	
No	695 (45.2)	486 (43.2)	209 (50.6)	
Own previous infection with SARS-CoV-2				0.80
Yes	19 (1.2)	15 (1.3)	4 (1.0)	
No	1518 (98.8)	1109 (98.7)	409 (99.0)	

^1^: Low: secondary modern school qualification (‘Haupt−/Volksschulabschluss’); ^2^: Intermediate: secondary school level I certificate (‘Mittlere Reife’); ^3^: High: general qualification for university entrance (‘Abitur’) or entrance qualification limited to universities of applied sciences (‘Fachhochschulreife’); ^a^: German profession ‘Rettungssanitäter’; ^b^: German profession ‘Rettungsassistent’; ^c^: German profession ‘Notfallsanitäter’; * refers to the third German sex “divers” (introduced by law); **Fisher’s Exact Test *p* < 0.05

As shown in Fig. 1, the majority of participants in both waves agreed that their personal risk of SARS-CoV-2 contraction was higher compared to the general population (87.0% first wave, 78.9% second wave). Major stressors were uncertainty about the temporal scope of the pandemic (82.0% first wave, 80.9% second wave) and one’s childcare situation (60.4% first wave, 69.3% second wave). Almost three quarters of EMS workers (69.9% first wave, 79.7% second wave) felt sufficiently prepared for the pandemic and only 18.8% (first wave) and 13.3% (second wave) felt burdened by their financial situation. The level of all stressors decreased from the first to second wave except for uncertainty about one’s childcare situation which increased. Pandemic-related attitudes implying preparedness became more positive between the two study points, that are, feeing prepared for dealing with SARS-CoV-2 patients, reporting sufficient PPE availability for personal use, and feeling sufficiently protected by PPE. Most of the observed changes were statistically significant (see Fig. 1).

### Logistic regression results

#### SARS-CoV-2 related attitudes

Logistic regression results for pandemic-related attitudes are displayed in Table 2. Male EMS workers were more likely to feel sufficiently prepared compared to non-male workers. EMS workers with intermediate education were more likely to report an increased pandemic-related workload and those with the highest education felt significantly more prepared than workers with lower education. The level of paramedic training also showed significant associations with an increase in workload: the group of workers with 520 h of training reported a significantly higher workload. Good self-rated health was significantly associated with a feeling of being sufficiently protected by available PPE and a feeling of being sufficiently prepared for dealing with SARS-CoV-2 patients. EMS workers who reported suspected or confirmed cases of SARS-CoV-2 among their colleagues were less likely to feel protected by PPE and to feel prepared for SARS-CoV-2. Participants classified as having depressive symptoms were less likely to feel prepared for SARS-CoV-2 and less likely to feel protected by PPE. Suspected or confirmed SARS-CoV-2 cases among colleagues were associated with reporting higher perceived odds of contraction and an increased workload. Participants with symptoms of anxiety significantly more frequently reported an increase in workload.

**Table 2:** Multivariable logistic regression results for SARS-CoV-2-related attitudes among emergency medical services workers (n = 1124)

	SARS-CoV-2 related attitudes			
	Higher perceived risk of contraction	Feeling of sufficient protection from infection	Feeling sufficiently prepared	Increased workload due to pandemic
	OR (95% CI)	OR (95% CI)	OR (95% CI)	OR (95% CI)
Sex				
Male (vs. other)	1.43 (0.92-2.23)	1.27 (0.91-1.78)	**1.72 (1.22-2.42)**	0.90 (0.65-1.27)
Age				
29-37 (vs. 18-28)	0.93 (0.58-1.49)	0.82 (0.59-1.14)	1.01 (0.72-1.47)	1.25 (0.89-1.75)
38 and older (vs. 18-28)	0.86 (0.52-1.41)	0.98 (0.69-1.40)	1.27 (0.87-1.87)	1.34 (0.94-1.92)
Permanent Partner				
Yes (vs. no)	0.87 (0.56-1.35)	0.82 (0.60-1.12)	0.81 (0.58-1.12)	1.34 (0.97-1.83)
Children under care in same household				
Yes (vs. no)	1.22 (0.78-1.91)	**0.65 (0.48-0.89)**	0.83 (0.56-1.16)	0.95 (0.69-1.29)
Highest level of education				
Intermediate^2^ (vs. low^1^)	0.80 (0.35-1.79)	1.19 (0.68-2.11)	1.47 (0.82-2.64)	**1.95 (1.06-3.59)**
High^3^ (vs. low^1^)	1.11 (0.49-2.51)	1.17 (0.66-2.06)	**1.97 (1.09-3.54)**	1.56 (0.84-2.87)
Highest level of paramedic training				
520 h training^a^ (vs. 3 years^c^)	0.89 (0.56-1.42)	0.81 (0.58-1.13)	0.92 (0.65-1.32)	**1.69 (1.21-2.35)**
2 years training^b^ (vs. 3 years^c^)	0.60 (0.35-1.03)	0.75 (0.49-1.14)	0.89 (0.56-1.39)	1.08 (0.70-1.68)
Self-rated health				
Good (vs. bad)	0.62 (0.28-1.40)	**1.83 (1.16-2.90)**	**1.85 (1.17-2.94)**	0.83 (0.53-1.32)
SARS-CoV-2 cases among friends and family				
Yes (vs. no)	0.83 (0.53-1.31)	1.11 (0.81-1.54)	0.82 (0.59-1.14)	0.98 (0.71-1.36)
SARS-CoV-2 cases among colleagues				
Yes (vs. no)	**1.63 (1.13-2.33)**	**0.69 (0.53-0.89)**	**0.62 (0.47-0.82)**	**1.63 (1.25-2.11)**
Depression				
Yes (vs. no)	1.18 (0.64-2.15)	**0.51 (0.35-0.75)**	**0.52 (0.35-0.77)**	1.20 (0.81-1.76)
Anxiety Disorder				
Yes (vs. no)	1.84 (0.99-3.44)	0.73 (0.51-1.04)	0.73 (0.50-1.06)	**2.46 (1.71-3.43)**

Statistically significant findings highlighted with bold letters; *OR* Odds ratio, *CI* Confidence interval; ^1^: Low: secondary modern school qualification (‘Haupt−/Volksschulabschluss’); ^2^: Intermediate: secondary school level I certificate (‘Mittlere Reife’); ^3^: High: general qualification for university entrance (‘Abitur’) or entrance qualification limited to universities of applied sciences (‘Fachhochschulreife’); ^a^: German profession ‘Rettungssanitäter’; ^b^: German profession ‘Rettungsassistent’; ^c^: German profession ‘Notfallsanitäter’

#### SARS-CoV-2 related stressors

Regression results for pandemic-related stressors are displayed in Tables 3 and 4. Male EMS workers were less likely to feel uncertain about correct behavior and less likely to feel burdened by their childcare situation. Thoughts about SARS-CoV-2 contraction at the workplace were less common among EMS workers with higher education than among those with lower education. Compared to the group with the longest paramedic training (i.e. 3 years), both groups with lower training reported an increased uncertainty about their financial situation. Increased odds of feeling burdened by a shortfall of colleagues were found for EMS workers with suspected or confirmed cases among colleagues, those of older age, with depressive symptoms, and anxiety symptoms. In contrast, those caring for children were less burdened by a shortfall of colleagues. Participants suffering from symptoms of anxiety significantly more frequently reported to be burdened by thoughts about SARS-CoV-2 contraction at the workplace, uncertainty about how to act correctly and uncertainty about contact persons. Participants with children under care living in the same household were more likely to feel uncertain about how to act correctly, uncertain about contact persons and uncertain about the temporal scope of the pandemic.

**Table 3:** Multivariable logistic regression results for SARS-CoV-2-related stressors among emergency medical services workers (n = 1124)

	SARS-CoV-2 related stressors			
	Thoughts about contraction at workplace	Shortfall of colleagues	Childcare situation*	Not being able to let patients down
	OR (95% CI)	OR (95% CI)	OR (95% CI)	OR (95% CI)
Sex				
Male (vs. other)	0.74 (0.53-1.04)	0.84 (0.60-1.18)	0.37 (0.14-0.95)	0.80 (0.57-1.10)
Age				
29-37 (vs. 18-28)	1.08 (0.77-1.50)	1.37 (0.98-1.93)	2.61 (0.82-8.35)	1.00 (0.72-1.38)
38 and older (vs. 18-28)	1.04 (0.73-1.47)	1.74 (1.22-2.49)	3.03 (0.96-9.57)	0.93 (0.66-1.31)
Permanent Partner				
Yes (vs. no)	1.19 (0.88-1.62)	1.43 (1.04-1.97)	1.90 (0.54-6.65)	1.26 (0.94-1.71)
Children under care in same household				
Yes (vs. no)	1.19 (0.87-1.63)	0.71 (0.52-0.98)	–	1.06 (0.78-1.44)
Highest level of education				
Intermediate^2^ (vs. low^1^)	0.41 (0.22-0.77)	1.00 (0.56-1.81)	0.54 (0.16-1.78)	0.93 (0.53-1.63)
High^3^ (vs. low^1^)	0.37 (0.20-0.69)	0.88 (0.49-1.57)	0.51 (0.15-1.67)	0.81 (0.46-1.42)
Highest level of paramedic training				
520 h training^a^ (vs.3 years^c^)	1.09 (0.79-1.52)	0.97 (0.69-1.37)	0.92 (0.42-2.01)	1.47 (1.06-2.03)
2 years training^b^ (vs.3 years^c^)	0.91 (0.59-1.40)	0.82 (0.53-1.29)	1.11 (0.48-2.57)	0.82 (0.54-1.26)
Self-rated health				
Good (vs. bad)	0.71 (0.43-1.17)	0.65 (0.41-1.04)	0.50 (0.21-1.19)	0.63 (0.39-1.01)
SARS-CoV-2 cases among friends and family				
Yes (vs. no)	0.96 (0.70-1.33)	1.27 (0.92-1.75)	0.96 (0.52-1.78)	1.22 (0.89-1.67)
SARS-CoV-2 cases among colleagues				
Yes (vs. no)	1.52 (1.18-1.96)	1.84 (1.41-2.39)	1.64 (1.00-2.70)	1.38 (1.08-1.78)
Depression				
Yes (vs. no)	1.52 (1.00-2.31)	1.78 (1.21-2.64)	0.87 (0.39-1.94)	1.55 (1.05-2.28)
Anxiety Disorder				
Yes (vs. no)	4.00 (2.60-6.16)	2.62 (1.81-3.79)	2.34 (1.09-5.00)	1.95 (1.35-2.82)

^*^only for *n* = 313 EMS workers with children under care in their household; Significant findings highlighted with bold letters; *OR* Odds ratio, *CI* Confidence interval; ^1^: Low: secondary modern school qualification (‘Haupt−/Volksschulabschluss’); ^2^: Intermediate: secondary school level I certificate (‘Mittlere Reife’); ^3^: High: general qualification for university entrance (‘Abitur’) or entrance qualification limited to universities of applied sciences (‘Fachhochschulreife’); ^a^: German profession ‘Rettungssanitäter’; ^b^: German profession ‘Rettungsassistent’; ^c^: German profession ‘Notfallsanitäter’

**Table 4:** Multivariable logistic regression results for SARS-CoV-2-related stressors among emergency medical services workers (n = 1124)

	SARS-CoV-2 related stressors			
	Uncertainty about acting correctly	Uncertainty about contact persons	Uncertainty about financial situation	Uncertainty about temporal scope
	OR (95% CI)	OR (95% CI)	OR (95% CI)	OR (95% CI)
Sex				
Male (vs. other)	0.56 (0.39-0.79)	0.72 (0.52-1.00)	1.05 (0.69-1.61)	0.81 (0.53-1.26)
Age				
29-37 (vs. 18-28)	0.88 (0.63-1.23)	0.89 (0.64-1.23)	1.03 (0.67-1.59)	0.66 (0.44-1.00)
38 and older (vs. 18-28)	0.74 (0.52-1.04)	0.88 (0.62-1.25)	1.06 (0.67-1.67)	0.72 (0.47-1.12)
Permanent Partner				
Yes (vs. no)	0.81 (0.60-1.10)	1.08 (0.80-1.46)	1.25 (0.84-1.86)	0.92 (0.63-1.34)
Children under care in same household				
Yes (vs. no)	1.60 (1.17-2.19)	1.54 (1.13-2.10)	1.06 (0.72-1.57)	1.73 (1.16-2.60)
Highest level of education				
Intermediate^2^ (vs. low^1^)	0.74 (0.41-1.32)	0.85 (0.48-1.50)	0.85 (0.45-1.60)	0.69 (0.31-1.55)
High^3^ (vs. low^1^)	0.64 (0.35-1.15)	0.73 (0.41-1.28)	0.53 (0.28-1.00)	0.60 (0.27-1.33)
Highest level of paramedic training				
520 h training^a^ (vs. 3 years^c^)	1.04 (0.75-1.44)	1.02 (0.74-1.42)	2.66 (1.79-3.94)	0.96 (0.63-1.45)
2 years training^b^ (vs. 3 years^c^)	1.22 (0.79-1.89)	1.19 (0.78-1.83)	1.92 (1.15-3.20)	1.00 (0.5971.71)
Self-rated health				
Good (vs. bad)	0.93 (0.57-1.51)	0.83 (0.52-1.32)	0.68 (0.41-1.16)	0.72 (0.36-1.44)
SARS-CoV-2 cases among friends and family				
Yes (vs. no)	1.28 (0.93-1.78)	1.68 (1.23-2.31)	1.06 (0.71-1.58)	0.83 (0.56-1.24)
SARS-CoV-2 cases among colleagues				
Yes (vs. no)	1.26 (0.98-1.63)	1.18 (0.92-1.52)	1.07 (0.77-1.49)	1.11 (0.80-1.51)
Depression				
Yes (vs. no)	2.43 (1.57-3.75)	2.28 (1.54-3.36)	1.58 (1.02-2.45)	2.09 (1.10-4.01)
Anxiety Disorder				
Yes (vs. no)	2.50 (1.66-3.76)	1.67 (1.16-2.40)	2.65 (1.77-3.98)	2.79 (1.47-5.29)

Significant findings highlighted with bold letters; *OR* Odds ratio, *CI* Confidence interval; ^1^: Low: secondary modern school qualification (‘Haupt−/Volksschulabschluss’); ^2^: Intermediate: secondary school level I certificate (‘Mittlere Reife’); ^3^: High: general qualification for university entrance (‘Abitur’) or entrance qualification limited to universities of applied sciences (‘Fachhochschulreife’); ^a^: German profession ‘Rettungssanitäter’; ^b^: German profession ‘Rettungsassistent’; ^c^: German profession ‘Notfallsanitäter’

#### SARS-CoV-2 related work outcomes

Participants in the oldest age group showed reduced odds of believing that care for patients with other diseases has been suffering (0.72 [0.53-0.98], see Additional file 2). EMS workers with only 520 h of training were less likely to report that sufficient amounts of PPE were available for them to use (0.66 [0.48-0.90]) (see Additional file 2).

### Sensitivity analysis

After removal of depressive symptoms and symptoms of anxiety from the multivariable models, the effect estimates changed only marginally (see Additional file 3). Pooling of data from both study waves for logistic regression yielded similar estimates (see Additional file 4).

## Discussion

To our knowledge, our study is the first to investigate attitudes and stressors related to the SARS-CoV-2 pandemic among EMS workers in Germany and the first study worldwide to examine stressors among this professional group at two separate time points during an infectious disease pandemic. We found moderate degrees of uncertainty during the peak of the pandemic in April 2020 (first wave) and found lower prevalences of pandemic-related stressors later in May 2020 (second wave). Most EMS workers agreed that their individual risk of SARS-CoV-2 contraction was higher compared to the general population. This is in accordance with studies among healthcare staff during previous infectious disease outbreaks [23–25]. The most common stressor among EMS workers in our study was uncertainty about the temporal scope of the pandemic which complies with Lee et al. (2005) who investigated stressors of Taiwanese nurses caring for SARS patients during the outbreak in 2003 [26]. Other stressors in our study were reported in moderate frequency and some, such as worries about financial matters and one’s workload, were reported to a rather limited extent. These findings differ from those reported by e.g., Vinck et al. (2011) who investigated public health workers in the Netherlands during the H1N1 pandemic in 2009 and found most of them to report an increase in workload [27]. The prevalences of depression and anxiety among our study sample are lower than those observed among anesthetist-intensivists in Italy during the COVID-19 pandemic [28], but are higher than those of Chinese frontline workers [29]. Differences in these prevalences could stem from different tools used to measure depression and anxiety symptoms among the three studies. Tan et al. (2020) argue that frontline staff might receive special psychological support and therefore suffer less from depression and anxiety [29]. Furthermore, the sample sizes of the three studies strongly differed, which implies varying precision related to the quantification of depression prevalences. Differing precision could in turn contribute to seemingly inconsistent estimations. Overall, our results indicate that EMS workers are less affected by existential fears and rather worry about their personal infection risk.

Although only one month had passed between the two study waves, first tendencies towards a possible psychological adaption to the pandemic situation could be observed. Agreement to items such as preparedness, availability of PPE and feeling of sufficient protection from PPE increased by over 10% points between both waves. These observations are paralleled by a decrease in most pandemic-related stressors. The only stressor that increased between both waves was the feeling of being burdened by one’s childcare situation. While it may have been possible for parents to compensate for the loss of childcare facilities in the short term, this may have become more and more challenging over time. Possibly, this is because the longer children stay at home, the greater the burden on parents becomes.

### Investigation of determinants

Except for comparison of our findings with the available evidence, we discussed logistic regression results thoroughly with experts from the German Association of EMS in order to also provide explanations of significant associations based on practical experience. This approach was chosen as only limited literature on stressors among EMS workers during a pandemic exists and the approach of this study was solely exploratory. Results indicated that older EMS workers felt more burdened by a pandemic-related shortfall of colleagues than younger colleagues. A shortfall of colleagues implies additional shifts for the remaining work force. Whereas younger EMS staff may be able to handle additional shifts (including night shifts) very well and can be attracted by additional payments, this might not hold true for older EMS workers. Additional shifts likely become more strenuous with increasing age and money might no longer serve as adequate incentive for older workers.

Reports of SARS-CoV-2 cases among colleagues were associated with a higher perceived risk of virus contraction, an increased perceived workload, increased thoughts about contraction at the workplace and feeling burdened by a shortfall of colleagues. EMS workers who have observed SARS-CoV-2 cases among their colleagues might feel more susceptible to contracting a SARS-CoV-2 infection due to close co-working with potentially infected colleagues or because they have seen how easily one may be infected. The increase in workload presumably stems from a lack of staff that leads to additional shifts for all remaining staff.

EMS workers with 520 h of training reported a higher workload and less availability of PPE compared to workers with highest EMS training. This may be explained as follows: in Germany, different ambulance types perform different services. Emergency medical technicians with 520 h training commonly man the type of ambulance that offers patient transports whereas paramedics with highest training usually man ambulances for rescue services. During the SARS-CoV-2 pandemic, rescue service utilization has dropped [30] but patient transports have increased (according to experts of the German Association of Emergency Medical Service). This may explain the increased workload and shortage in PPE for EMS workers with 520 h of training. This finding is consistent with observations from the SARS outbreak in 2004 when Ko et al. (2004) found non-SARS related ambulance activities to decrease in Taiwan [31].

### Strengths and limitations

Our study captured attitudes and stressors during the early peak of the SARS-CoV-2 pandemic in Spring 2020 and therefore with minimal potential for recall bias. We conducted two independent surveys within five weeks and were therefore able to capture short-term changes in the perception of the pandemic. The comparison of participant characteristics between the first and second wave revealed no significant differences, indicating that our sample provided a good representation of members and followers of the German Association of EMS. Nonetheless, several limitations to our study must be discussed. Firstly, no exact response rate could be calculated due to the online distribution of the questionnaire. Secondly, compared to the official numbers of the federal employment agency, fewer female EMS workers took part in our study (16.7% vs. 27.0%) and the age group of 25-54 years was overrepresented in the study (80.4% vs. 65.70%) [17]. Younger employees might not be as likely to follow the activities of the German Association of EMS. The online distribution of the survey may have not reached older employees that do not engage in online activities. A third limitation is that our survey is not able to detect regional differences in Germany as different legislations and circumstances apply in each federal state. In some states rescue service tasks are taken over mainly by a professional fire brigade, whereas in other states also voluntary helpers perform rescue service tasks. These differences may affect working conditions, headcounts, and commitment and consequently also affect perceived attitudes and stressors during the SARS-CoV-2 pandemic. Due to the study’s cross-sectional design no causal relationships but only associations can be reported which is a further limitation. Finally, we used a self-devised questionnaire with unknown psychometric properties as no validated items on pandemic-related attitudes and stressors of medical staff were available. However, validity was increased due to the close discussion with experts of the field regarding comprehensibility and completeness of items. These experts have not only worked as EMS workers themselves but are in daily contact with thousands of EMS workers following the German Association of EMS.

### Recommendations based on the study findings

Our study findings suggest that EMS workers in Germany feel burdened by their childcare situation. Therefore, political decision-making processes should consider this point to a greater extent and childcare opportunities for healthcare staff should be guaranteed. Secondly, as most EMS workers rather feel burdened by the risk of an own infection than by existential fears, sufficient PPE supplies have to be held in stock in cases of future infectious disease outbreaks. Although we found moderate levels of agreement to different types of stressors, future training opportunities should be provided to all EMS workers to prepare in the best possible way for a pandemic situation. In the past, the important role of the employer has been highlighted [6, 9] and intervention programs among EMS workers have shown to be able to not only increase knowledge, but also behavioral intentions regarding use of PPE and willingness to work during a pandemic situation [27, 28]. Longitudinal studies are needed for the identification of causal predictors of attitudes and stressors among EMS workers during the SARS-CoV-2 pandemic. Future studies may also shed light on the implications of pandemic-related stressors on quality of patient care.

## Conclusions

In summary, this is the first study to provide in-depth data on attitudes, stressors and work-outcomes among EMS workers during the SARS-CoV-2 outbreak. We found moderate agreement to different stressors indicating that employees do not suffer from existential fears but are rather worried about their own risk of infection. We found all stressors but the childcare situation to decrease between both study waves indicating an adaption to the circumstances within a 5-week period. Finally, we identified subgroups at special risk to be burdened by the pandemic such as older employees, those with SARS-CoV-2 cases among their colleagues, and those with 520 h of training.

## Supplementary Information

**Additional file 1.** Items of the English language study questionnaire.

**Additional file 2.** Multivariable logistic regression results for SARS-CoV-2-related work outcomes among emergency medical services workers.

**Additional file 3.** Sensitivity analysis results: Logistic regression results without adjusting for depression and anxiety.

**Additional file 4.** Sensitivity analysis results: Logistic regression results after pooling data from both study waves.

## Data Availability

The datasets generated and/or analysed during the current study are available in the Zenodo repository, 10.5281/zenodo.4415689

## Abbreviations

CI: Confidence Interval
EMS: Emergency Medical Services
GAD: Generalized Anxiety Disorder Questionnaire
OR: Odds Ratio
PHQ: Patient Health Questionnaire
PPE: Personal Protective Equipment
