# Supplementary material for: Attitudes and stressors related to the SARS-CoV-2 pandemic among emergency medical services workers in Germany: a cross-sectional study
Source: BMC Health Serv Res. 2021 Aug 21;21:851. doi: 10.1186/s12913-021-06779-5 (PMC8380100; doi:10.1186/s12913-021-06779-5)
Supplement: Supplementary file 1 — Additional file 1. Items of the English language study questionnaire. [file 12913_2021_6779_MOESM1_ESM.pdf]

Questionnaire on the SARS-CoV-2 ("Coronavirus") pandemic

1. **Sex**
    - Male
    - Female
    - Non-binary

2. **Year of birth**
    - ___________

3. **Do you have a permanent partner?**
    - Yes
    - No

4. **Do you have persons in your household who need care?**
    - Yes, children under care
    - Yes, adults under care
    - Yes, both, children and adults under care
    - No

5. **What is your highest level of education?**
    - Secondary modern school qualification
    - Secondary school level I certificate
    - General qualification for university entrance or entrance qualification limited to universities of applied sciences
    - Other (e.g. obtained abroad)

6. **What is you highest paramedic training?**
    - 3 years training (“Notfallsanitäter/in”)
    - 2 years training (“Rettungsassistent/in”)
    - 520 hours training (“Rettungssanitäter/in”)
    - 320 hours training (“Rettungshelfer/in”)
    - None of the above

7. **Are you currently working as emergency medical services worker?**
    - Yes
    - No, working as other: _______________________
    - I am currently not employed (e.g. looking for work, maternity leave)

8. **In general, how would you describe your state of health?**
    - Very good
    - Good
    - Moderate
    - Bad
    - Very bad

9. **There are suspected or confirmed SARS-CoV-2 cases among my friends and family.**
    - Yes
    - No

10. **There are suspected or confirmed SARS-CoV-2 cases among my colleagues.**
    - Yes
    - No

11. You yourself have already tested positive for SARS-CoV-2.

- Yes
- No

The following questions refer to the current SARS-CoV-2 pandemic ("coronavirus"). Please indicate to what extent you agree with the following statements.

	Strongly Disagree	Disagree	Agree	Strongly agree
I feel sufficiently prepared for dealing with SARS-CoV-2 patients by my employer				
The risk of contracting SARS-CoV-2 is higher for me than for a person of same age and sex from the general population				
My workload has increased due to the SARS-CoV-2 pandemic				
Due to the SARS-CoV-2 pandemic the care for patients with other diseases has been suffering				
At my work all necessary materials for personal protection from SARS-CoV-2 are sufficiently available for me				
I can use materials for personal protection at my work so that I feel sufficiently protected from contracting SARS-CoV-2				
I am burdened with thoughts of a possible infection with SARS-CoV-2 during work hours				
I am burdened by the crisis-related shortfall of colleagues/staff at work				
I am burdened by the care situation of my children				
I am burdened by uncertainty about how to act correctly during the crisis				
I am burdened by uncertainty about contact persons during the crisis				
I am burdened by uncertainty about my financial situation during the crisis				
I am burdened by uncertainty about the temporal scope of the crisis				
I am burdened by a feeling of not being able to let patients down during the crisis				

Over the <u>last 2 weeks</u>, how often have you been bothered by the following problems?

	Not at all	Several days	More than half the days	Nearly every day
Feeling nervous, anxious or on edge				
Not being able to stop or control worrying				
Little interest or pleasure in doing things				
Feeling down, depressed or hopeless				
