# Supplementary material for: Attitudes and stressors related to the SARS-CoV-2 pandemic among emergency medical services workers in Germany: a cross-sectional study
Source: BMC Health Serv Res. 2021 Aug 21;21:851. doi: 10.1186/s12913-021-06779-5 (PMC8380100; doi:10.1186/s12913-021-06779-5)
Supplement: Supplementary file 2 — Additional file 2. Multivariable logistic regression results for SARS-CoV-2-related work outcomes among emergency medical services workers. [file 12913_2021_6779_MOESM2_ESM.pdf]

Supplementary Table 5. Multivariable logistic regression results for SARS-CoV-2-related work outcomes among emergency medical services workers (n=1124).

	SARS-CoV-2 related work outcomes	
	Suffering care of patients with other diseases	Sufficiently available material for personal protection
	OR (95% CI)	OR (95% CI)
Sex		
Male (vs. other)	0.87 (0.64-1.20)	1.23 (0.90-1.69)
Age		
29-37 (vs. 18-28)	0.90 (0.67-1.21)	0.82 (0.60-1.10)
38 and older (vs. 18-28)	**0.72 (0.53-0.98)**	0.78 (0.58-1.06)
Highest level of paramedic training		
520 hours training[a] (vs.3 years[c])	1.24 (0.91-1.69)	0.70 (0.46-1.05)
2 years training[b] (vs.3 years[c])	1.18 (0.79-1.78)	**0.66 (0.48-0.90)**

Significant findings highlighted with bold letters; OR odds ratio; CI confidence interval; a: German profession ‘Rettungssanitäter’; b: German profession ‘Rettungsassistent’; c: German profession ‘Notfallsanitäter’
