# Supplementary material for: Attitudes and stressors related to the SARS-CoV-2 pandemic among emergency medical services workers in Germany: a cross-sectional study
Source: BMC Health Serv Res. 2021 Aug 21;21:851. doi: 10.1186/s12913-021-06779-5 (PMC8380100; doi:10.1186/s12913-021-06779-5)
Supplement: Supplementary file 3 — Additional file 3. Sensitivity analysis results: Logistic regression results without adjusting for depression and anxiety. [file 12913_2021_6779_MOESM3_ESM.pdf]

Supplementary Table 1. Sensitivity Analysis: Multivariable Logistic regression results for SARS-CoV-2 related attitudes without adjusting for depression and anxiety disorder

	SARS-CoV-2 related attitudes			
	Higher perceived risk of contraction	Feeling of sufficient protection from infection	Feeling sufficiently prepared	Increased workload due to pandemic
	OR (95% CI)	OR (95% CI)	OR (95% CI)	OR (95% CI)
Sex				
Male (vs. other)	1.37 (0.88-2.13)	1.35 (0.97-1.87)	**1.81 (1.29-2.53)**	0.86 (0.62-1.19)
Age				
29-37 (vs. 18-28)	0.94 (0.59-1.51)	0.83 (0.59-1.15)	1.03 (0.73-1.46)	1.26 (0.91-1.76)
38 and older (vs. 18-28)	0.85 (0.52-1.40)	1.00 (0.71-1.43)	1.30 (0.89-1.90)	1.31 (0.92-1.86)
Permanent Partner				
Yes (vs. no)	0.85 (0.55-1.32)	0.84 (0.62-1.14)	0.83 (0.60-1.15)	1.27 (0.93-1.73)
Children under care in same household				
Yes (vs. no)	1.22 (0.78-1.92)	**0.66 (0.49-0.89)**	0.84 (0.60-1.16)	0.97 (0.71-1.31)
Highest level of education				
Intermediate[2] (vs. low[1])	0.78 (0.35-1.74)	1.28 (0.73-2.23)	1.57 (0.89-2.79)	1.76 (0.97-3.19)
High[3] (vs. low[1])	1.05 (0.47-2.37)	1.29 (0.74-2.24)	**2.15 (1.21-3.83)**	1.36 (0.75-2.47)
Highest level of paramedic training				
520 hours training[a] (vs. 3 years[c])	0.86 (0.54-1.38)	0.87 (0.62-1.20)	0.99 (0.70-1.40)	**1.58 (1.14-2.20)**
2 years training[b] (vs. 3 years[c])	0.61 (0.36-1.04)	0.73 (0.48-1.11)	0.86 (0.55-1.35)	1.07 (0.70-1.64)
Self-rated health				
Good (vs. bad)	0.55 (0.25-1.22)	**2.19 (1.40-3.41)**	**2.23 (1.42-3.48)**	0.69 (0.44-1.07)
SARS-CoV-2 cases among friends and family				
Yes (vs. no)	0.84 (0.54-1.33)	1.07 (0.78-1.48)	0.79 (0.57-1.10)	1.02 (0.75-1.40)
SARS-CoV-2 cases among colleagues				
Yes (vs. no)	**1.67 (1.16-2.39)**	**0.67 (0.52-0.86)**	**0.60 (0.45-0.79)**	**1.70 (1.31-2.20)**

Significant findings highlighted with bold letters; OR odds ratio; CI confidence interval; 1: Low: secondary modern school qualification (‘Haupt-/Volksschulabschluss’); 2: Intermediate: secondary school level I certificate (‘Mittlere Reife’); 3: High: general qualification for university entrance (‘Abitur’) or entrance qualification limited to universities of applied sciences (‘Fachhochschulreife’); a: German profession ‘Rettungssanitäter’; b: German profession ‘Rettungsassistent’; c: German profession ‘Notfallsanitäter’

Supplementary Table 2. Sensitivity Analysis: Multivariable Logistic regression results for SARS-CoV-2 related stressors without adjusting for depression and anxiety disorder

	SARS-CoV-2 related stressors			
	Thoughts about contraction at workplace	Shortfall of colleagues	Childcare situation*	Not being able to let patients down
	OR (95% CI)	OR (95% CI)	OR (95% CI)	OR (95% CI)
Sex				
Male (vs. other)	**0.70 (0.51-0.97)**	0.78 (0.56-1.08)	**0.36 (0.14-0.90)**	0.75 (0.55-1.04)
Age				
29-37 (vs. 18-28)	1.10 (0.80-1.52)	1.37 (0.98-1.90)	2.49 (0.79-7.83)	1.01 (0.73-1.39)
38 and older (vs. 18-28)	1.02 (0.72-1.43)	**1.65 (1.17-2.34)**	2.83 (0.91-8.79)	0.92 (0.65-1.29)
Permanent Partner				
Yes (vs. no)	1.23 (0.84-1.51)	1.35 (0.99-1.83)	1.64 (0.48-5.62)	1.22 (0.91-1.64)
Children under care in same household				
Yes (vs. no)	1.19 (0.88-1.62)	0.73 (0.54-1.00)	-	1.06 (0.79-1.44)
Highest level of education				
Intermediate[2] (vs. low[1])	**0.40 (0.22-0.74)**	0.92 (0.52-1.62)	0.51 (0.16-1.61)	0.88 (0.50-1.52)
High[3] (vs. low[1])	**0.35 (0.19-0.64)**	0.77 (0.44-1.35)	0.47 (0.15-1.50)	0.73 (0.42-1.27)
Highest level of paramedic training				
520 hours training[a] (vs.3 years[c])	1.02 (0.74-1.41)	0.91 (0.65-1.26)	0.87 (0.40-1.88)	1.38 (1.00-1.90)
2 years training[b] (vs.3 years[c])	0.90 (0.60-1.37)	0.85 (0.55-1.30)	1.16 (0.51-2.65)	0.83 (0.55-1.26)
Self-rated health				
Good (vs. bad)	**0.56 (0.35-0.89)**	**0.50 (0.32-0.79)**	**0.41 (0.18-0.95)**	**0.52 (0.33-0.82)**
SARS-CoV-2 cases among friends and family				
Yes (vs. no)	1.01 (0.74-1.38)	1.31 (0.96-1.80)	0.95 (0.51-1.75)	1.25 (0.92-1.71)
SARS-CoV-2 cases among colleagues				
Yes (vs. no)	**1.59 (1.24-2.04)**	**1.91 (1.48-2.47)**	**1.71 (1.05-2.80)**	**1.44 (1.12-1.84)**

Significant findings highlighted with bold letters; OR odds ratio; CI confidence interval; 1: Low: secondary modern school qualification (‘Haupt-/Volksschulabschluss’); 2: Intermediate: secondary school level I certificate (‘Mittlere Reife’); 3: High: general qualification for university entrance (‘Abitur’) or entrance qualification limited to universities of applied sciences (‘Fachhochschulreife’); a: German profession ‘Rettungssanitäter’; b: German profession ‘Rettungsassistent’; c: German profession ‘Notfallsanitäter’

Supplementary Table 2 (continued). Sensitivity Analysis: Multivariable Logistic regression results for SARS-CoV-2 related stressors without adjusting for depression and anxiety disorder

	SARS-CoV-2 related stressors			
	Uncertainty about acting correctly	Uncertainty about contact persons	Uncertainty about financial situation	Uncertainty about temporal scope
	OR (95% CI)	OR (95% CI)	OR (95% CI)	OR (95% CI)
Sex				
Male (vs. other)	**0.53 (0.38-0.74)**	**0.68 (0.49-0.93)**	0.94 (0.62-1.41)	0.76 (0.50-1.17)
Age				
29-37 (vs. 18-28)	0.89 (0.64-1.22)	0.89 (0.64-1.22)	1.05 (0.69-1.60)	0.67 (0.45-1.01)
38 and older (vs. 18-28)	0.73 (0.52-1.02)	0.86 (0.61-1.22)	1.03 (0.66-1.60)	0.71 (0.46-1.09)
Permanent Partner				
Yes (vs. no)	0.79 (0.59-1.06)	1.05 (0.78-1.41)	1.16 (0.79-1.71)	0.89 (0.61-1.30)
Children under care in same household				
Yes (vs. no)	**1.56 (1.15-2.12)**	**1.52 (1.12-2.05)**	1.09 (0.74-1.59)	**1.72 (1.15-2.57)**
Highest level of education				
Intermediate[2] (vs. low[1])	0.68 (0.39-1.21)	0.79 (0.45-1.37)	0.77 (0.42-1.43)	0.66 (0.30-1.46)
High[3] (vs. low[1])	0.57 (0.32-1.00)	0.65 (0.37-1.13)	**0.46 (0.25-0.87)**	0.54 (0.25-1.20)
Highest level of paramedic training				
520 hours training[a] (vs. 3 years[c])	0.95 (0.69-1.32)	0.95 (0.69-1.31)	**2.34 (1.60-3.43)**	0.90 (0.59-1.35)
2 years training[b] (vs. 3 years[c])	1.21 (0.79-1.85)	1.21 (0.80-1.83)	**1.89 (1.15-3.08)**	0.99 (0.59-1.69)
Self-rated health				
Good (vs. bad)	0.69 (0.44-1.09)	0.65 (0.42-1.01)	**0.52 (0.31-0.85)**	0.56 (0.28-1.11)
SARS-CoV-2 cases among friends and family				
Yes (vs. no)	1.33 (0.97-1.82)	**1.71 (1.26-2.33)**	1.11 (0.75-1.63)	0.88 (0.59-1.30)
SARS-CoV-2 cases among colleagues				
Yes (vs. no)	**1.32 (1.03-1.69)**	1.23 (0.96-1.58)	1.16 (0.84-1.61)	1.15 (0.84-1.57)

Significant findings highlighted with bold letters; OR odds ratio; CI confidence interval; 1: Low: secondary modern school qualification (‘Haupt-/Volksschulabschluss’); 2: Intermediate: secondary school level I certificate (‘Mittlere Reife’); 3: High: general qualification for university entrance (‘Abitur’) or entrance qualification limited to universities of applied sciences (‘Fachhochschulreife’); a: German profession ‘Rettungssanitäter’; b: German profession ‘Rettungsassistent’; c: German profession ‘Notfallsanitäter’
