# Supplementary material for: Attitudes and stressors related to the SARS-CoV-2 pandemic among emergency medical services workers in Germany: a cross-sectional study
Source: BMC Health Serv Res. 2021 Aug 21;21:851. doi: 10.1186/s12913-021-06779-5 (PMC8380100; doi:10.1186/s12913-021-06779-5)
Supplement: Supplementary file 4 — Additional file 4. Sensitivity analysis results: Logistic regression results after pooling data from both study waves. [file 12913_2021_6779_MOESM4_ESM.pdf]

Supplementary Table 3. Sensitivity Analysis: Multivariable Logistic Regression Results for SARS-CoV-2-related Attitudes (pooled data from first and second study wave, n=1537)

	SARS-CoV-2 related attitudes			
	Higher perceived risk of contraction	Feeling of sufficient protection from infection	Feeling sufficiently prepared	Increased workload due to pandemic
	OR (95% CI)	OR (95% CI)	OR (95% CI)	OR (95% CI)
Sex				
Male (vs. other)	1.28 (0.88-1.86)	1.23 (0.92-1.65)	**1.68 (1.24-2.28)**	0.77 (0.58-1.04)
Age				
29-37 (vs. 18-28)	1.10 (0.76-1.61)	0.82 (0.61-1.09)	0.96 (0.70-1.31)	**1.35 (1.01-1.82)**
38 and older (vs. 18-28)	0.98 (0.66-1.45)	1.02 (0.75-1.38)	1.03 (0.74-1.44)	**1.48 (1.09-2.02)**
Permanent Partner				
Yes (vs. no)	0.94 (0.66-1.33)	0.82 (0.63-1.07)	0.85 (0.63-1.14)	**1.37 (1.04-1.81)**
Children under care in same household				
Yes (vs. no)	1.06 (0.74-1.51)	**0.76 (0.58-0.99)**	0.83 (0.62-1.11)	0.91 (0.69-1.19)
Highest level of education				
Intermediate[2] (vs. low[1])	0.72 (0.37-1.39)	1.33 (0.82-2.13)	1.38 (0.84-2.28)	1.55 (0.94-2.57)
High[3] (vs. low[1])	0.97 (0.50-1.88)	1.23 (0.77-1.98)	**1.69 (1.02-2.79)**	1.26 (0.76-2.09)
Highest level of paramedic training				
520 hours training[a] (vs. 3 years[c])	1.03 (0.70-1.50)	0.75 (0.56-1.00)	0.88 (0.64-1.20)	**1.54 (1.15-2.05)**
2 years training[b] (vs. 3 years[c])	0.71 (0.45-1.13)	0.80 (0.55-1.15)	0.84 (0.56-1.25)	1.02 (0.69-1.50)
Self-rated health				
Good (vs. bad)	**0.50 (0.26-0.99)**	**1.67 (1.14-2.45)**	**1.68 (1.14-2.49)**	0.99 (0.67-1.46)
SARS-CoV-2 cases among friends and family				
Yes (vs. no)	0.82 (0.57-1.19)	1.06 (0.78-1.40)	0.76 (0.57-1.03)	0.97 (0.73-1.28)
SARS-CoV-2 cases among colleagues				
Yes (vs. no)	**1.73 (1.29-2.31)**	**0.65 (0.52-0.81)**	**0.65 (0.51-0.83)**	**1.66 (1.33-2.08)**
Depression				
Yes (vs. no)	1.07 (0.68-1.71)	**0.56 (0.41-0.77)**	**0.52 (0.38-0.73)**	1.35 (0.97-1.86)
Anxiety Disorder				
Yes (vs. no)	**1.77 (1.07-2.92)**	**0.68 (0.50-0.93)**	**0.61 (0.44-0.84)**	**2.21 (1.62-3.02)**

Significant findings highlighted with bold letters; OR odds ratio; CI confidence interval; 1: Low: secondary modern school qualification (‘Haupt-/Volksschulabschluss’); 2: Intermediate: secondary school level I certificate (‘Mittlere Reife’); 3: High: general qualification for university entrance (‘Abitur’) or entrance qualification limited to universities of applied sciences (‘Fachhochschulreife’); a: German profession ‘Rettungssanitäter’; b: German profession ‘Rettungsassistent’; c: German profession ‘Notfallsanitäter’

Supplementary Table 4. Sensitivity Analysis: Multivariable Logistic regression results for SARS-CoV-2 related stressors (pooled data from first and second study wave, n=1537)

	SARS-CoV-2 related stressors			
	Thoughts about contraction at workplace	Shortfall of colleagues	Childcare situation*	Not being able to let patients down
	OR (95% CI)	OR (95% CI)	OR (95% CI)	OR (95% CI)
Sex				
Male (vs. other)	0.86 (0.64-1.16)	0.95 (0.70-1.28)	**0.28 (0.12-0.66)**	0.81 (0.61-1.08)
Age				
29-37 (vs. 18-28)	1.06 (0.80-1.40)	**1.53 (1.14-2.06)**	**2.64 (1.02-6.84)**	0.95 (0.72-1.25)
38 and older (vs. 18-28)	1.09 (0.81-1.47)	**1.92 (1.41-2.61)**	**2.63 (1.03-6.72)**	0.86 (0.64-1.15)
Permanent Partner				
Yes (vs. no)	1.23 (0.95-1.60)	**1.36 (1.03-1.79)**	1.41 (0.49-4.10)	1.23 (0.95-1.59)
Children under care in same household				
Yes (vs. no)	1.06 (0.81-1.38)	**0.63 (0.48-0.83)**	-	1.14 (0.88-1.48)
Highest level of education				
Intermediate[2] (vs. low[1])	**0.57 (0.35-0.92)**	1.19 (0.73-1.96)	0.84 (0.33-2.13)	0.88 (0.55-1.41)
High[3] (vs. low[1])	**0.56 (0.34-0.92)**	0.99 (0.60-1.63)	0.80 (0.31-2.02)	0.83 (0.52-1.32)
Highest level of paramedic training				
520 hours training[a] (vs.3 years[c])	1.00 (0.76-1.33)	1.00 (0.74-1.35)	0.73 (0.36-1.51)	**1.42 (1.07-1.87)**
2 years training[b] (vs.3 years[c])	0.94 (0.65-1.36)	0.91 (0.62-1.34)	0.99 (0.48-2.06)	0.89 (0.62-1.29)
Self-rated health				
Good (vs. bad)	0.82 (0.55-1.24)	0.70 (0.47-1.03)	0.61 (0.29-1.29)	0.70 (0.47-1.02)
SARS-CoV-2 cases among friends and family				
Yes (vs. no)	0.99 (0.75-1.31)	1.23 (0.92-1.63)	1.12 (0.64-1.94)	1.08 (0.82-1.42)
SARS-CoV-2 cases among colleagues				
Yes (vs. no)	**1.60 (1.29-1.99)**	**1.85 (1.47-2.32)**	1.35 (0.88-2.06)	**1.33 (1.08-1.65)**
Depression				
Yes (vs. no)	**1.73 (1.23-2.45)**	**2.18 (1.57-3.03)**	0.96 (0.48-1.92)	**1.65 (1.20-2.27)**
Anxiety Disorder				
Yes (vs. no)	**4.08 (2.83-5.87)**	**2.43 (1.76-3.34)**	**2.42 (1.22-4.79)**	**2.07 (1.51-2.84)**

*only for n=424 EMS workers with children under care in their household; Significant findings highlighted with bold letters; OR odds ratio; CI confidence interval; 1: Low: secondary modern school qualification (‘Haupt-/Volksschulabschluss’); 2: Intermediate: secondary school level I certificate (‘Mittlere Reife’); 3: High: general qualification for university entrance (‘Abitur’) or entrance qualification limited to universities of applied sciences (‘Fachhochschulreife’); a: German profession ‘Rettungssanitäter’; b: German profession ‘Rettungsassistent’; c: German profession ‘Notfallsanitäter’

Supplementary Table 4 (continued). Sensitivity Analysis: Multivariable Logistic regression results for SARS-CoV-2 related stressors (pooled data from first and second study wave, n=1537)

	SARS-CoV-2 related stressors			
	Uncertainty about acting correctly	Uncertainty about contact persons	Uncertainty about financial situation	Uncertainty about temporal scope
	OR (95% CI)	OR (95% CI)	OR (95% CI)	OR (95% CI)
Sex				
Male (vs. other)	**0.65 (0.48-0.87)**	**0.73 (0.55-0.98)**	1.15 (0.78-1.69)	0.89 (0.61-1.29)
Age				
29-37 (vs. 18-28)	0.98 (0.74-1.29)	0.96 (0.72-1.27)	1.05 (0.72-1.52)	0.73 (0.51-1.03)
38 and older (vs. 18-28)	0.79 (0.59-1.07)	0.86 (0.64-1.16)	0.96 (0.64-1.42)	0.72 (0.50-1.05)
Permanent Partner				
Yes (vs. no)	0.84 (0.65-1.09)	0.98 (0.75-1.27)	1.38 (0.97-1.96)	0.96 (0.69-1.32)
Children under care in same household				
Yes (vs. no)	**1.37 (1.05-1.78)**	**1.45 (1.11-1.89)**	1.07 (0.76-1.51)	**1.54 (1.09-2.16)**
Highest level of education				
Intermediate[2] (vs. low[1])	0.95 (0.59-1.54)	0.85 (0.53-1.36)	0.84 (0.49-1.43)	0.92 (0.50-1.70)
High[3] (vs. low[1])	0.85 (0.53-1.37)	0.68 (0.42-1.09)	**0.48 (0.28-0.84)**	0.82 (0.45-1.51)
Highest level of paramedic training				
520 hours training[a] (vs. 3 years[c])	1.04 (0.78-1.37)	0.99 (0.74-1.31)	**2.48 (1.75-3.52)**	0.86 (0.61-1.22)
2 years training[b] (vs. 3 years[c])	1.21 (0.83-1.76)	1.25 (0.86-1.81)	**1.91 (1.22-2.99)**	0.91 (0.58-1.45)
Self-rated health				
Good (vs. bad)	0.83 (0.55-1.25)	0.77 (0.52-1.13)	**0.61 (0.39-0.95)**	0.70 (0.39-1.27)
SARS-CoV-2 cases among friends and family				
Yes (vs. no)	1.28 (0.97-1.70)	**1.57 (1.19-2.06)**	0.94 (0.65-1.34)	0.85 (0.60-1.19)
SARS-CoV-2 cases among colleagues				
Yes (vs. no)	1.23 (0.99-1.53)	1.18 (0.95-1.47)	1.25 (0.94-1.67)	1.14 (0.87-1.49)
Depression				
Yes (vs. no)	**2.15 (1.51-3.06)**	**2.13 (1.54-2.95)**	1.46 (1.00-2.14)	**2.23 (1.30-3.82)**
Anxiety Disorder				
Yes (vs. no)	**2.95 (2.06-4.20)**	**1.86 (1.36-2.55)**	**2.32 (1.61-3.34)**	**2.71 (1.56-4.69)**

Significant findings highlighted with bold letters; OR odds ratio; CI confidence interval; 1: Low: secondary modern school qualification (‘Haupt-/Volksschulabschluss’); 2: Intermediate: secondary school level I certificate (‘Mittlere Reife’); 3: High: general qualification for university entrance (‘Abitur’) or entrance qualification limited to universities of applied sciences (‘Fachhochschulreife’); a: German profession ‘Rettungssanitäter’; b: German profession ‘Rettungsassistent’; c: German profession ‘Notfallsanitäter’
